# Utilizing Novel Lignocellulosic Material from Hart’s-Tongue Fern (*Asplenium scolopendrium*) Leaves for Crystal Violet Adsorption: Characterization, Application, and Optimization

**DOI:** 10.3390/polym15193923

**Published:** 2023-09-28

**Authors:** Giannin Mosoarca, Cosmin Vancea, Simona Popa, Mircea Dan, Sorina Boran

**Affiliations:** Faculty of Industrial Chemistry and Environmental Engineering, Politehnica University Timisoara, Bd. V. Parvan, No. 6, 300223 Timisoara, Romania; giannin.mosoarca@upt.ro (G.M.); cosmin.vancea@upt.ro (C.V.); simona.popa@upt.ro (S.P.)

**Keywords:** low-cost bioadsorbent, cationic dye, adsorption mechanism, Taguchi method

## Abstract

In this work, a new lignocellulosic adsorbent was obtained and tested for crystal violet dye removal from water. The material was obtained from hart’s-tongue fern (*Asplenium scolopendrium*) leaves after minimal processing, without chemical or thermal treatment. The surface of the material was characterized using a variety of techniques, including FTIR, SEM, and color analysis. The effect of various factors on the adsorption capacity was then investigated and discussed. The kinetic and equilibrium studies showed that the general-order kinetic model and the Sips isotherm are the most suitable to describe the adsorption process. The equilibrium time was reached after 20 min and the maximum calculated value of the adsorption capacity was 224.2 (mg g^−1^). The determined values for the thermodynamic parameters indicated physical adsorption as the main mechanism involved in the process. The Taguchi method was used to optimize the adsorption conditions and identify the most influential controllable factor, which was pH. ANOVA (general linear model) was used to calculate the percentage contribution of each controllable factor to dye removal efficiency. Analysis of all the results shows that hart’s-tongue fern (*Asplenium scolopendrium*) leaves are a very inexpensive, readily available, and effective adsorbent for removing crystal violet dye from aqueous solutions.

## 1. Introduction

In contemporary times, the utilization of chemical dyes has witnessed a notable surge across a spectrum of industries including textiles, paper, plastics, printing, tanning, pigments, food, and pharmaceuticals. This proliferation, while catering to diverse industrial needs, has concurrently raised substantial concerns regarding its ecological ramifications presenting serious risks to the environment due to the inevitable leakage of effluents containing dangerous substances for soil and natural waters [1,2,3,4].

In addition to their well-documented harmful effects on health, the presence of dyes in aquatic environments leads to water discoloration and reduces oxygen levels, causing significant harm to aquatic life. This is especially concerning as most dyes used in industries are synthetic and contain aromatic rings, making their degradation in the environment a slow process. Moreover, the degradation of these synthetic dyes can produce secondary toxic substances that further damage the environment. Consequently, the effective removal of these hazardous dyes from industrial wastewater is an urgent issue, leading to the protection of aquatic ecosystems and also carries economic benefits [1,2,3,4,5,6,7].

Crystal violet, a versatile dye used in a wide range of industries, including textiles, printing, and pharmaceuticals, is also used in human and veterinary medicine [8,9]. However, crystal violet is a toxic and non-biodegradable dye, and its presence in residual effluents can have a detrimental impact on the health of aquatic ecosystems and the photosynthesis of aquatic plants. Even though it poses little threat to human health, it can still have negative consequences such as increased heart rate, eye irritation, respiratory problems, cyanosis, kidney failure, and cancer. Therefore, treatment of wastewater before discharge to remove the dye is imperative [4,7,8].

Numerous physical, chemical, electrochemical, and biological techniques are commonly used to remove dyes from waters: chemical precipitation, microbial degradation, coagulation/flocculation, advanced oxidation processes, membrane techniques, electrochemical treatments, and adsorption. However, the industrial application of many of these methods are often limited due to their high cost, energy consumption, and operation time. Additionally, some methods can generate large amounts of secondary sludge that require supplementary treatment. To select the best removal method, it is important to consider not only the process efficiency but also the raw materials and associated costs in order to achieve a balance between environmental and economic considerations. Among these, adsorption has been identified as one of the most effective, being primarily preferred due to its low cost, ease of use, simple design, adaptability, and high separation efficiency even at low dye concentrations [1,2,5,6,7,8,9,10,11,12,13,14,15,16,17,18,19].

Many operating variables influence adsorption efficiency, including contaminant concentration, adsorbent dosage, pH, contact time, adsorbate–adsorbent interaction, and adsorbent type. As a result, there is a need for new adsorbents that are widely available, reusable, and cost-effective. Due to their clean, biodegradable, and rotatable qualities, lignocellulosic biomasses have attracted special attention in obtaining adsorbent materials. These materials are mainly composed of macromolecules, especially lignocellulose, which includes cellulose, lignin, and hemicellulose. These materials possess a number of interesting properties such as: abundance, renewability, high porosity, and very low costs. In addition, they have favorable mechanical properties and are easily modifiable. As a result, these materials represent a significant resource for the production of eco-friendly bio-polymeric adsorbents with a high adsorption capacity [20,21,22,23,24].

Hart’s-tongue fern (*Asplenium scolopendrium*) is a fern species that grows in Europe, North America, North Africa, and East Asia, either as a terrestrial plant or as an epiphyte on rotting and moist bark. It is a perennial, evergreen plant that prefers shady and humid areas, with leaves having a length of 40–70 cm and a width of 8–12 cm. In addition to its exceptional beauty, it also has numerous pharmaceutical attributes, being used in traditional medicine to treat lung and biliary problems. It has diuretic, astringent, and anticancer effects, being used to treat wounds, seborrhea, and acne [25,26].

The objective of this paper was to propose the hart’s-tongue fern (*Asplenium scolopendrium*) leaves as a new adsorbent for crystal violet dye from aqueous solutions. The surface characteristics of the adsorbent were investigated using scanning electron microscopy (SEM), Fourier transform infrared spectroscopy (FTIR), and color analysis. Equilibrium, kinetics, and thermodynamic parameters were calculated and analyzed to elucidate the adsorption process mechanism. The Taguchi method was used to optimize the parameters that influenced the adsorption process to obtain maximum dye removal efficiency.

## 2. Materials and Methods

The mature leaves of hart’s-tongue fern, dried and shredded, were purchased from a company whose activity is the processing and packaging of medicinal plants, StefMar (Ramnicu Valcea, Romania). These were grounded using an electric mill; the resulting powder was then washed with distilled water to remove color and turbidity. The wet material was dried in a laboratory oven at 105 °C for 24 h.

In order to determine the characteristics of the adsorbent surface, Fourier transform infrared spectroscopy (FTIR), scanning electron microscopy (SEM), and color analysis (in CIEL*a*b* system) were used and the point of zero charge (pH_PZC_) was determined. FTIR analysis was performed with a Shimadzu Prestige-21 FTIR spectrophotometer (Shimadzu, Kyoto, Japan) using a pellet obtained by pressing a mixture of KBr with the adsorbent powder. SEM analysis was carried out with a Quanta FEG 250 microscope (FEI, Eindhoven, The Netherlands) at 800 × magnification and the color analysis with a Cary-Varian 300 Bio UV-VIS colorimeter (Varian Inc., Mulgrave, Australia) under D65 (natural light) illumination and with 10 observer angles. The point of zero charge (pH_PZC_) was calculated according to the solid addition method [27] using a WTW Ino-lab pH-meter (model 7310, Xylem Analytics Germany, Weilheim, Germany). The granulometric analysis of the powder adsorbent was carried out according to standard ASTM D6913-04(2009)e1 [28].

All adsorption experiments were carried out in batch mode using three independent replicates, using a volume of dye solution of 50 mL. The values of the main parameters that affect the process varied as follows: pH = 2–12, stirring time = 1–60 min, initial dye concentration = 25–700 (mg L^−1^), adsorbent dose = 1–6 (g L^−1^), temperature = 285–319 K, and ionic strength = 0–0.25 (mol L^−1^). Dilute solutions of HCl 0.1 (mol dm^−3^) and NaOH 0.1 (mol dm^−3^) were used for pH adjustment and the ionic strength was modified by adding solid NaCl. The crystal violet concentration was measured with a Specord 200 PLUS UV-VIS spectrophotometer (Analytik Jena, Jena, Germany) at a wavelength of 590 nm.

In all the experimental determinations carried out, a control sample was also used in which the adsorbent material was not introduced. The absorbance and concentration of the control sample remained practically constant throughout the determinations, which proves that there are no degradation processes of the dye in the solution.

Equilibrium and kinetics of adsorption were analyzed by modeling the experimental data using several adsorption isotherms and kinetic models. In the Supplementary Materials, Table S1, these isotherms and models along with their corresponding non-linear equations are presented [29,30]. In order to establish which isotherm and kinetic model is the most appropriate to characterize the dye adsorption process on adsorbent material obtained from hart’s-tongue fern, the values for determination coefficient (R^2^), sum of square error (SSE), chi-square (χ^2^), and average relative error (ARE) were calculated. The calculation equations of these parameters are detailed in the Supplementary Materials, Table S2 [30].

The main adsorption mechanism was identified based on the thermodynamic parameters, calculated with the equations described in Supplementary Materials, Table S3 [29].

The Taguchi method, a powerful experimental design technique, was used to optimize the critical factors influencing the adsorption process, maximizing the removal efficiency of the dye. For this purpose, the L27 orthogonal array with six factors at three levels was used and the signal-to-noise ratio (S/N) was analyzed to assess the experimental results.

ANOVA (general linear model) analysis was used to evaluate the Taguchi method results and to calculate the percentage contribution of each controllable factor on the crystal violet removal efficiency. The required mathematical calculations were conducted with the Minitab 19 Software (version 19.1.1, Minitab LLC, State College, PA, USA).

In the desorption study, the dye-loaded adsorbent was stirred continuously for two hours with three different desorbing agents: distilled water, HCl (0.1 M), and NaOH (0.1 M) and then the amount of the dye released into the solution was measured. The equation used to calculate the desorption efficiency is shown in the Supplementary Materials, Table S4.

## 3. Results and Discussion

### 3.1. Adsorbent Surface Characterisation

**Figure 1:**
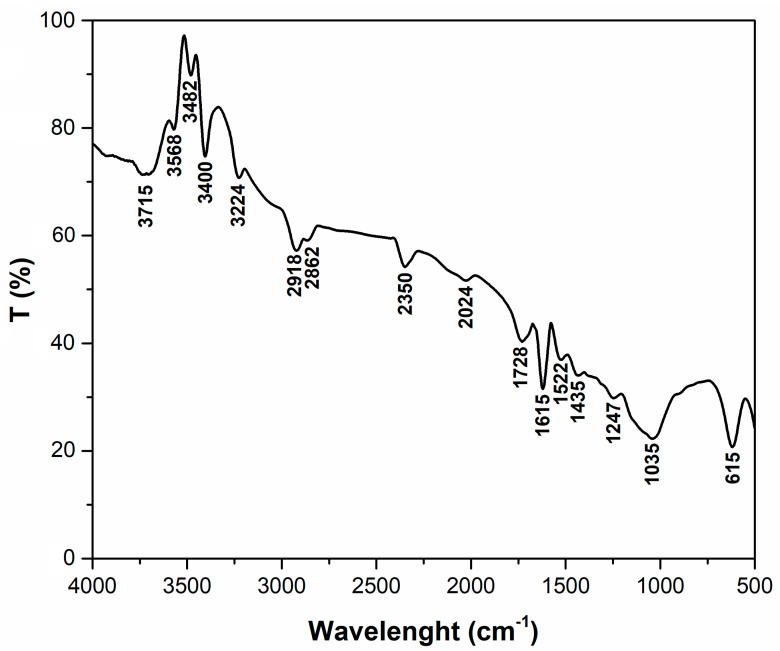
The FTIR spectra of adsorbent obtained from hart’s-tongue fern (*Asplenium scolopendrium*) leaves.

Figure 1 shows the FTIR spectrum for adsorbent material obtained from hart’s-tongue fern. The analysis of the peaks highlighted in this spectrum (Table 1) indicates that they belong to functional groups that can interact with crystal violet dye; these groups are specific to the main constituents of the adsorbent, namely, cellulose, hemicellulose, and lignin. The correspondence of the peaks identified with the specific functional groups is as follows: 3715 cm^−1^—OH-stretching mode of the free OH of water [31], 3568 cm^−1^, 3482 cm^−1^, and 3400 cm^−1^—OH- stretching vibration related to cellulose and hemicellulose [32,33,34]; 3224 cm^−1^—N−H stretching [35]; 2918 cm^−1^ –CH stretching vibration in cellulose and hemicellulose [36]; 2862 cm^−1^—CH_2_ asymmetric stretching [37]; 2350 cm^−1^ and 2024 cm^−1^—stretching of aromatic ring C=C [38,39]; 1728 cm^−1^—C=O stretching vibration of the carboxylic group from lignin and hemicellulose [40]; 1615 cm^−1^—stretching vibration of carboxyl group [41]; 1522 cm^−1^—aromatic C=C/C=N stretching [42]; 1435 cm^−1^—CH bending in lignin [43]; 1247 cm^−1^—C–O stretching vibration in lignin [44]; 1035 cm^−1^—C-O, C=C, and C-C-O stretching in cellulose, hemicellulose, and lignin [45], and 615 cm^−1^—the vibration elongation of C-H bonds in aromatics [46].

**Figure 2:**
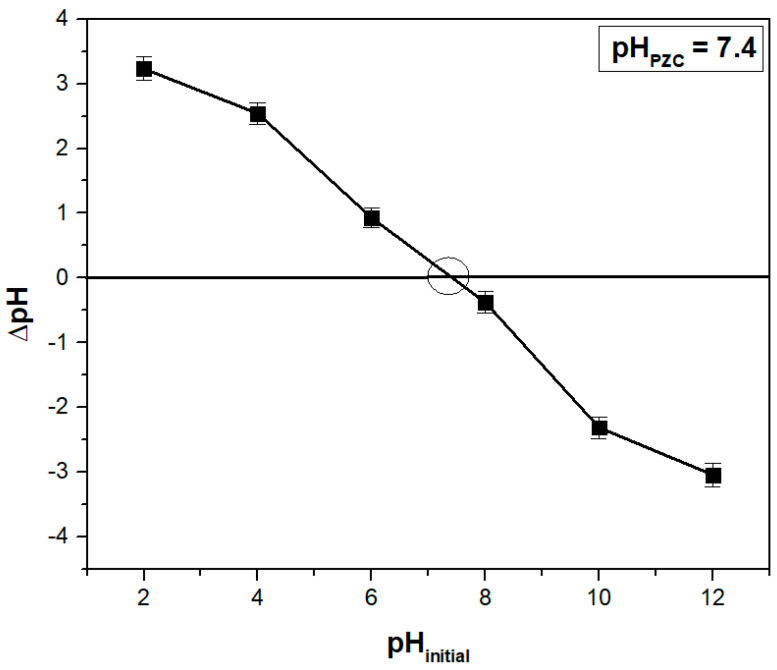
Determination of point of zero charge (pH_PZC_) for the studied adsorbent using the solid addition method.

Another critical parameter in adsorption studies is the point of zero charge (pH_PZC_), which is the pH at which the net surface charge of an adsorbent material is zero. At pH values higher than pH_PZC_, the surface is negatively charged, favoring the adsorption of cationic dyes, while at lower values, the effect is the opposite [19,47]. The value determined for the adsorbent obtained from hart’s-tongue fern leaves was 7.4 (Figure 2), being comparable to the values determined in other studies for similar adsorbents such as 6.98 for weeping willow (*Salix babylonica*) leaves [47], 7.1 for chicory (*Cichorium intybus*) leaves [48], 7.5 for gulmohar (*Delonix regia*) leaves [49], and 7.7 boxwood (*Buxus sempervirens*) leaves [50].

Figure S1, in the Supplementary Materials, shows the granulometric distribution of the hart’s-tongue fern leaves powder. As the dry leaves are brittle and easy to grind, the granulometric distribution of the powder is very narrow after grinding with an average particle size of 0.056 mm.

**Figure 3:**
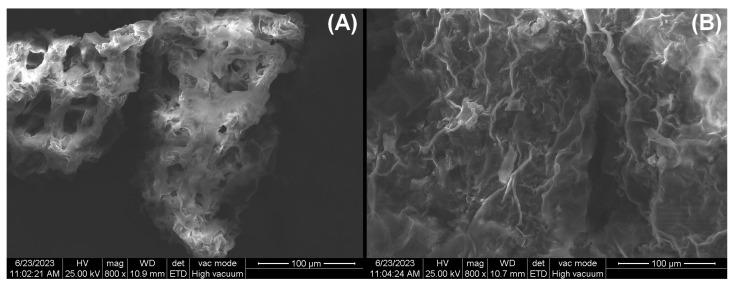
SEM images of adsorbent obtained from hart’s-tongue fern (*Asplenium scolopendrium*) leaves: (**A**) Before adsorption and (**B**) After adsorption.

Scanning electron microscopy (SEM) images of the adsorbent surface before and after dye adsorption are shown in Figure 3. Initially, the surface is heterogeneous, with many irregularities and different pores, which may provide a large number of adsorption sites (Figure 3A). After adsorption, the surface is more compact and uniform because the adsorbed dye molecules fill and cover the pores and irregularities (Figure 3B).

**Figure 4:**
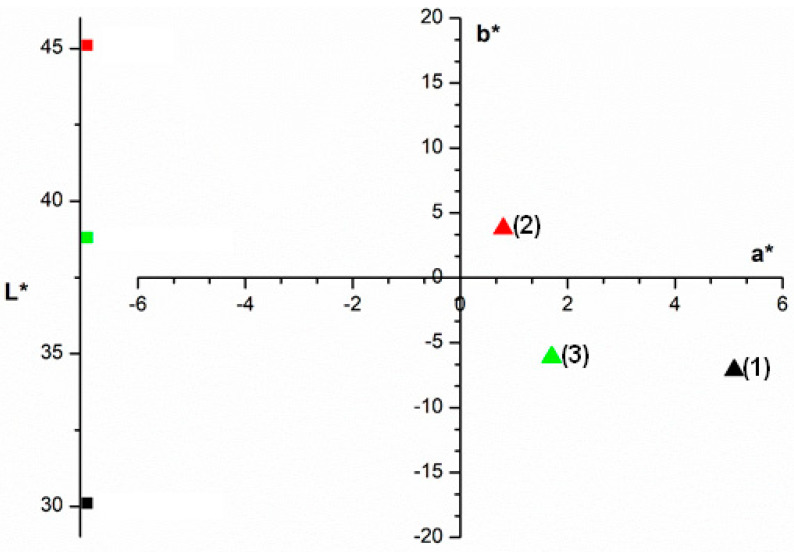
Color analysis for the adsorbent material, before and after adsorption, using CIEL*a*b* color parameters of: (1) Crystal violet dye, (2) Adsorbent before adsorption, and (3) Adsorbent after adsorption.

Figure 4 shows the CIELab* color analysis results for the studied adsorbent before and after dye adsorption. The analysis reveals changes in the L*, a*, and b* parameters after the adsorption of crystal violet dye. Point (1) represents the color of the cationic dye, and points (2) and (3) represent the color of the adsorbent before and after adsorption, respectively. The point representing the adsorbent color moves towards the color quadrant of the crystal violet dye after adsorption, indicating that the adsorbent color is modified by the dye adsorption.

### 3.2. The Influence of pH, Ionic Strength, and Adsorbent Dose on Adsorption Capacity

**Figure 5:**
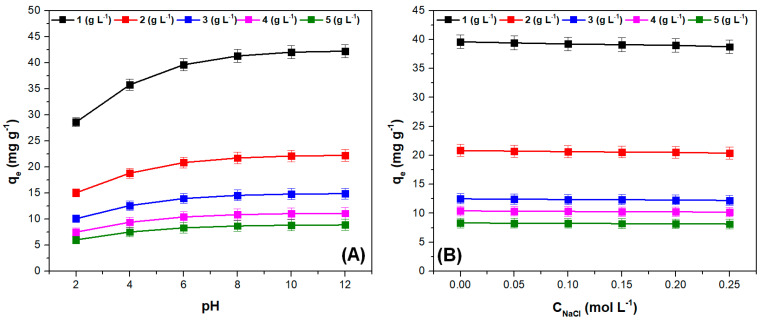
The influence of pH (**A**) and ionic strength (**B**), on adsorption capacity, at different adsorbent doses (*Adsorption conditions:* (**A**) contact time = 20 min, adsorbent dose = 2 (g L^−1^), crystal violet concentration = 50 (mg L^−1^), temperature = 295 K, ionic strength = 0 (mol L^−1^); (**B**) pH = 6, others identical to (**A**)).

The influence of pH and ionic strength on adsorption capacity at different adsorbent doses is illustrated in Figure 5. As expected, at pH values higher than pH_PZC_, the adsorption capacity had the highest values, highlighting the positive effect of the electrostatic attraction between the adsorbate surface and the cationic dye [51,52]. Increasing the adsorbent dose increases the number of available adsorption sites, but a significant portion of these sites remained unsaturated. This fact, along with the agglomeration of the adsorbent particles that can appear when the dose increases, lead to a decrease of the adsorption capacity [46,53,54]. Similar effects of pH and adsorbent dose were reported in other studies where the same type of adsorbent materials were used to retain crystal violet dye [51,55,56,57]. The increase in ionic strength results in a negligible decrease in adsorption capacity, indicating the adsorbent material’s affinity for the cationic dye.

While increasing the ionic strength of the solution may introduce competition between crystal violet cations and sodium ions for the occupation of adsorption sites on the adsorbate surface, this does not significantly affect the retention of the dye. This suggests that the adsorption process is not solely driven by electrostatic attraction. This is further supported by the observation that the adsorption capacity does not increase significantly at pH values above the point of zero charge (pH_PZC_).

These results indicate that the proposed adsorbent material is suitable for practical applications, such as the removal of cationic dyes from wastewaters containing other salts together with the considered dye.

### 3.3. Equilibrum Isotherms

The adsorption equilibrium was examined by modeling the experimental data using the isotherm models described in Table 1, which lists the isotherms’ constants and the corresponding error parameter. The information in the table indicates that the Sips isotherm best describes the process, this isotherm having the highest value for R^2^ and the lowest values for SSE, χ^2^ and ARE.

**Figure 6:**
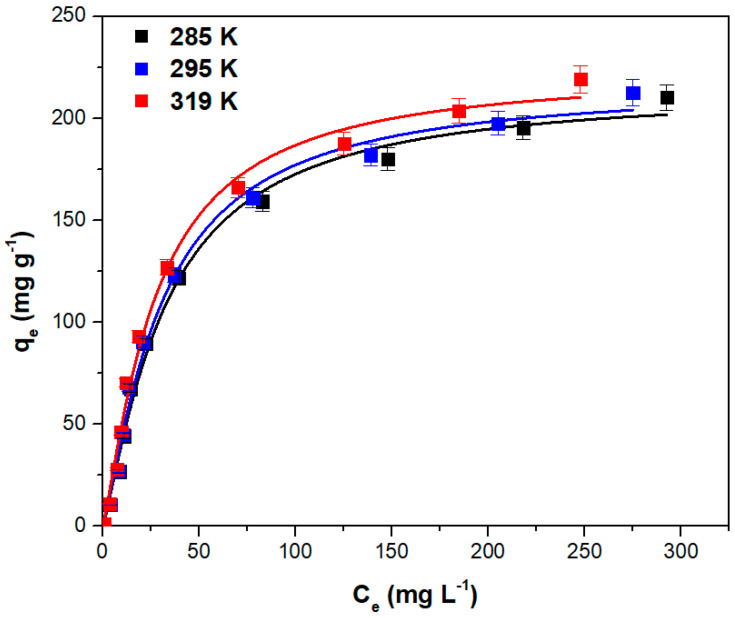
The experimental data points and the fitted Sips isotherm curves at various temperatures (*Adsorption conditions*: pH = 6, contact time = 20 min, adsorbent dose = 2 (g L^−1^), ionic strength = 0 (mol L^−1^)).

Figure 6 depicts the fitted Sips isotherm curves at various temperatures. According to the figure, the adsorption capacity increases with temperature. The solutions’ viscosity decreases as the temperature rises, therefore the mobility of the dye molecules increases. This has a beneficial impact on the adsorption capacity with the process being endothermic [58,59,60].

The maximum absorption capacities of various similar adsorbents obtained from plant leaves and used for crystal violet adsorption are compared in Table 2. The data indicate that the adsorbent obtained from hart’s-tongue fern (*Asplenium scolopendrium*) leaves has an adsorption capacity higher than other adsorbents, demonstrating the usefulness of the new adsorbent proposed in this work.

### 3.4. Kinetic Study

**Figure 7:**
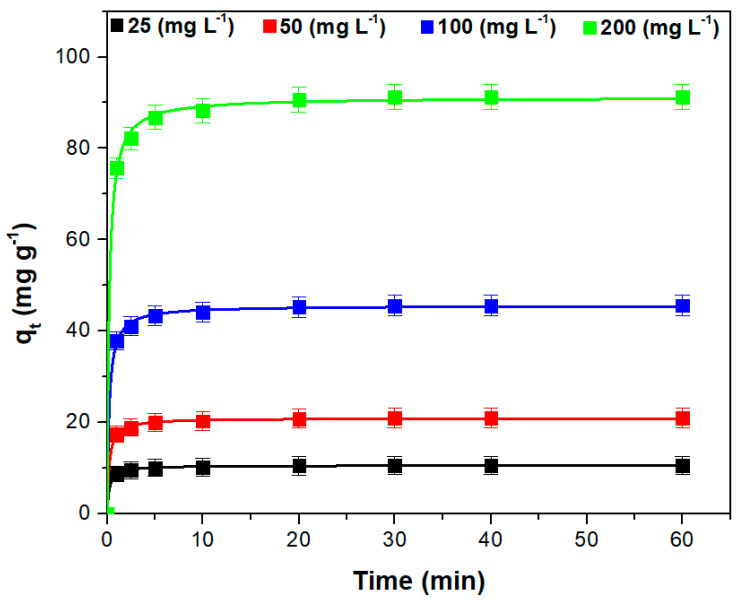
The experimental data points and the fitted general-order kinetic model at various initial dye concentration (Adsorption conditions: pH = 6, adsorbent dose = 2 (g L^−1^), temperature = 295 K, ionic strength = 0 (mol L^−1^)).

The effect of contact time on the ability of the adsorbent material to retain the dye at different initial dye concentrations is illustrated in Figure 7. The adsorption capacity increases quickly in the first minutes, due to a large number of adsorption sites accessible for the dye retention, and then more slowly as it approaches equilibrium. This is reached after 20 min when it is assumed that the adsorbent surface is almost completely coated by dye molecules [55,60]. The obtained equilibrium time is lower than those reported in the scientific literature for other adsorbents based on plant leaves (Table 3).

Increasing the initial concentration of the dye has a positive effect on the adsorption capacity due to the increase in the concentration gradient and the number of collisions between the dye molecules and the adsorbent material particles [8,46,51,53,55].

Five kinetic models were tested to model the experimental results. Table 4 presents these models together with their constants and the corresponding error parameter. The highest value for R^2^ and the lowest values for SSE, χ^2^, and ARE indicate that the general- order model is the proper model to describe the crystal violet adsorption. Figure 7 shows the fitted curves of this model at various initial dye concentrations. In the scientific literature, it is mentioned that the general-order kinetic model characterizes the adsorption of the crystal violet dye on similar adsorbent materials such as motherwort biomass [67] and sour cherry leaf [68].

### 3.5. Thermodynamic Study

The thermodynamic parameters listed in Table 5 were calculated from the slope and intercept of the plot of ln K_L_ versus 1/T, which is shown in Figure S2 of the Supplementary Materials, based on experimental data collected at three different temperatures: 282, 293, and 307 K. The standard Gibbs free energy change (ΔG^0^) is negative and varies with increasing temperature, indicating that the adsorption process is spontaneous and favorable. The standard enthalpy change (ΔH^0^) and standard entropy change (ΔS^0^) are both positive, indicating that the adsorption process is endothermic and increases randomness at the solid–liquid interface [55,58].

The value of ∆H^0^ lower than 20 (kJ mol^−1^) indicates that the main mechanism is physical adsorption, with van der Waals interaction implied in the process [69,70]. The standard Gibbs free energy change (ΔG^0^) of the adsorption process is between −80 and −20 (kJ mol^−1^), but closer to −20 (kJ mol^−1^). This suggests that there is a small chemical effect that may enhance the adsorption [43,71].

### 3.6. Optimization Using the Taguchi Method

The Taguchi method was used to determine the optimal adsorption conditions, which were based on an L27 orthogonal array experimental design. The six controllable factors that formed the basis of this array, together with their levels, are shown in Table 6.

The Taguchi method is a powerful way to design experiments. It focuses on finding the signal-to-noise ratio (S/N), which is a measure of how accurate and reliable the results are. The S/N ratio is calculated by comparing the response to the noise, which is any factor that can affect the accuracy of the results.

The Taguchi method has two main advantages: it minimizes the number of experiments needed and it provides a visual representation of the best conditions.

In this study, the Taguchi method was used to improve the efficiency of dye removal. The “larger is the better” option for the S/N ratio was used [72,73,74]. Table 7 details the L27 orthogonal array experimental design, the results of the experiments, and the S/N ratio for each experiment.

Table 8 shows the signal-to-noise (S/N) ratios for each factor at each level and their significant ranks. These ratios indicate how much each factor affects the effectiveness of dye removal. The higher the S/N ratio, the greater the impact of the factor. Based on the S/N ratios and significant ranks, pH has the most impact on dye removal efficiency, while temperature has the least. The Taguchi approach leads to the following optimal adsorption conditions: pH of 12, contact time of 60 min, adsorbent dose of 6 (g L^−1^), initial dye concentration of 200 (mg L^−1^), temperature of 319 K, and ionic strength of 0.0 (mol L^−1^). Table 8 also shows the ANOVA analysis results and the contribution percent of each controllable factor on crystal violet removal efficiency. Their value indicates the same hierarchy of influence of the controllable factors as the Taguchi technique.

**Figure 8:**
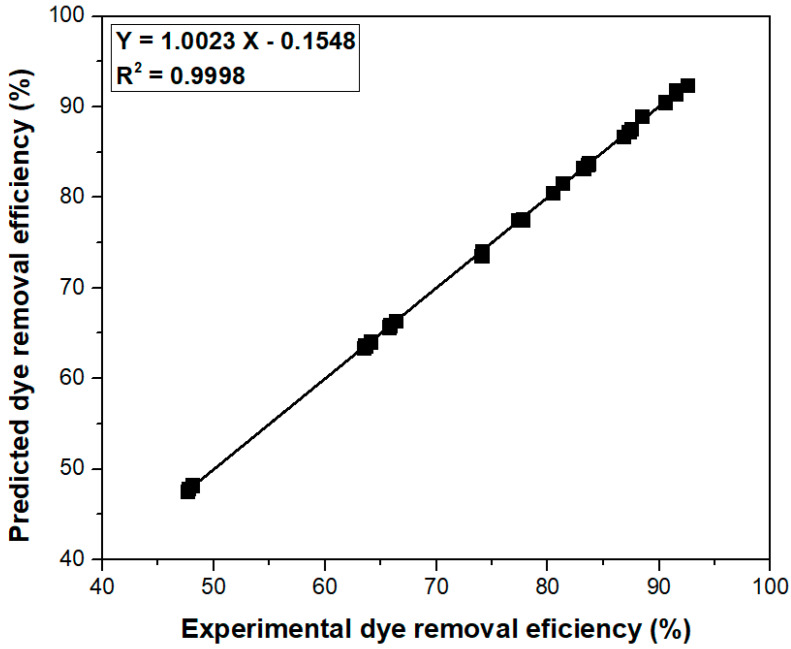
The relationship between the dye removal efficiency values measured in the experiment and those predicted by the Taguchi method.

By correlating the experimental dye removal efficiency values to those predicted by optimization, the validity of the Taguchi experimental design was confirmed (Figure 8). The value of determination coefficient R^2^ obtained for linear regression demonstrates a high degree of accuracy of the Taguchi approach.

### 3.7. Desorption Study

The desorption of crystal violet dye from the absorbent material was inefficient, regardless of the desorbing agent used, suggesting that it is not practical to reuse it. The desorption efficiencies were 7.83%, 29.38%, and 17.35% for distilled water, HCl, and NaOH, respectively. However, the low cost and abundance of hart’s-tongue fern leaves offset this drawback. Additionally, the absorbent material can be incinerated to generate energy, which is a simple and efficient way to reuse it.

## 4. Conclusions

Within this study, a novel lignocellulosic adsorbent was proposed to remove crystal violet dye from water. The source material for this adsorbent was derived from the leaves of the hart’s-tongue fern (*Asplenium scolopendrium*), having undergone a procedure of minimal processing that deliberately avoided both chemical and thermal treatments.

FTIR analysis identified different functional groups specific to the main constituents of the adsorbent (cellulose, hemicellulose, and lignin) that can interact with the crystal violet dye. SEM and color analysis, before and after adsorption, revealed changes in the morphology and color of the adsorbent, confirming the retention of the dye on its surface. The augmentation of specific parameters, such as pH, contact time, initial dye concentration, and temperature, exerts a favorable impact on the enhancement of the adsorption capacity value. The increase of the ionic strength resulted in a nearly negligible reduction in the adsorption capacity, underscoring the inherent affinity of the adsorbent towards crystal violet dye. Sips isotherm was the most suitable to characterize the process compared to other isotherms tested: Langmuir, Freundlich, Temkin, Sips, and Redlich–Peterson. The adsorbent exhibits an adsorption capacity (224.2 mg g^−1^) surpassing that of comparable adsorbents, suggesting the efficacy and practical value of the new proposed adsorbent. Equilibrium is reached after 20 min and the general-order model is the most proper model to describe the crystal violet adsorption. The thermodynamic parameters indicate a spontaneous and favorable process, the main mechanism being physical adsorption, with van der Waals interaction involved, along with a small chemical effect that may enhance adsorption. The Taguchi method and ANOVA analysis were used to determine the best conditions for adsorption (using an L27 orthogonal array experimental design) and the relative importance of each controllable factor on the removal efficiency of crystal violet, respectively. pH had the greatest impact on dye removal efficiency (75.84%), while temperature had the least (0.22%). The Taguchi method had good accuracy, with a good match between the experimental dye removal efficiency values and those predicted by the optimization.

The comprehensive assessment of the acquired data suggests that the hart’s-tongue fern (*Asplenium scolopendrium*) leaves serve as a cost-effective, readily accessible, and efficient adsorbent for eliminating crystal violet dye from aqueous solutions.

## Figures and Tables

**Table 1:** The constants and the corresponding error parameters for the tested adsorption isotherms.

Isotherm Model	Parameters	Value		
		285 K	295 K	319 K
Langmuir	K_L_ (L mg^−1^)	0.023 ± 0.004	0.025 ± 0.003	0.028 ± 0.003
	q_max_ (mg g^−1^)	237.2 ± 9.1	239.8 ± 8.4	247.3 ± 9.2
	R^2^	0.9916	0.9916	0.9915
	χ^2^	13.64	13.78	14.21
	SSE	557.2	569.5	605.4
	ARE (%)	13.15	13.14	13.16
Freundlich	K_f_ (mg g^−1^)	21.82 ± 3.21	22.06 ± 2.74	22.74 ± 0.92
	1/n	0.41 ± 0.07	0.41 ± 0.05	0.42 ± 0.04
	R^2^	0.9446	0.9446	0.9944
	χ^2^	51.18	51.73	53.33
	SSE	3401	3476	3694
	ARE (%)	22.32	22.34	22.35
Temkin	K_T_ (L mg^−1^)	0.265 ± 0.043	0.265 ± 0.048	0.266 ± 0.057
	b (kJ g^−1^)	48.85 ± 5.43	43.32 ± 2.41	46.87 ± 4.81
	R^2^	0.9923	0.9923	0.9923
	χ^2^	24.15	24.38	25.16
	SSE	451.5	464.2	493.4
	ARE (%)	30.99	30.94	30.97
Sips	Q_sat_ (mg g^−1^)	215.1 ± 8.4	217.5 ± 5.3	224.2 ± 7.3
	K_S_ (L mg^−1^)	0.014 ± 0.003	0.014 ± 0.002	0.016 ± 0.002
	n	1.22 ± 0.15	1.23 ± 0.14	1.24 ± 0.16
	R^2^	0.9939	0.9939	0.9939
	χ^2^	6.99	7.06	7.29
	SSE	371.7	379.7	403.5
	ARE (%)	10.02	10.02	10.03
Redlich–Peterson	K_RP_ (L g^−1^)	5.54	5.60 ± 0.84	5.78
	a_RP_ (L mg^−1^)	0.016	0.016 ± 0.003	0.017
	Β_RP_	1.05	1.05 ± 0.09	1.06
	R^2^	0.9918	0.9918	0.9918
	χ^2^	12.19	12.32	12.70
	SSE	524.7	536.3	569.4
	ARE (%)	12.72	12.71	12.72

**Table 2:** The maximum absorption capacities of various similar adsorbents obtained from plant leaves and used for crystal violet adsorption.

Adsorbent	Maximum Adsorption Capacity (mg g^−1^)	Reference
hart’s-tongue fern (*Asplenium scolopendrium*) leaves	224.2	This study
lilac tree leaf	196.7	[61]
pineapple leaf	78.22	[51]
jackfruit leaf	43.39	[62]
*Syzygium cumini* leaves	38.75	[63]
date palm leaves	37.73	[52]
*Platanus orientalis* leaf	25.88	[64]
*Adiantum capillus-veneris* leaves	18.51	[4]
*Calligonum comosum* leaf	5.00	[65]
*Calotropis procera* leaf	4.14	[66]

**Table 3:** The equilibrium times reported in the scientific literature for various adsorbents based on plant leaves and used for crystal violet adsorption.

Adsorbent	Equilibrium Time (min)	Reference
hart’s-tongue fern (*Asplenium scolopendrium*) leaves	20	This study
lilac tree leaf	40	[61]
date palm leaves	21	[52]
*Platanus orientalis* leaf	30	[64]
*Syzygium cumini* leaves	60	[63]
*Calotropis procera* leaf	60	[66]
*Adiantum capillus-veneris* leaves	90	[4]
jackfruit leaf	120	[62]

**Table 4:** The constants and the corresponding error parameters for the tested kinetic models.

Kinetic Model	Parameters	Initial Dye Concentration (mg L^−1^)			
		25	50	100	200
Pseudo-first order	k_1_ (min^−1^)	1.82 ± 0.21	1.83 ± 0.24	1.81 ± 0.31	1.82 ± 0.34
	q_e,calc_ (mg g^−1^)	10.33 ± 0.27	20.45 ± 0.62	44.33 ± 1.78	89.04 ± 3.75
	R^2^	0.9916	0.9912	0.9914	0.9915
	χ^2^	0.07	0.16	0.34	0.68
	SSE	0.78	3.21	14.88	58.93
	ARE (%)	13.38	13.51	13.41	13.39
Pseudo-second order	k_2_ (min^−1^)	0.42 ± 0.08	0.21 ± 0.03	0.09 ± 0.02	0.04 ± 0.01
	q_e,calc_ (g mg^−1^ min^−1^)	10.58 ± 0.34	20.93 ± 0.82	45.60 ± 1.34	91.16 ± 4.62
	R^2^	0.9991	0.9988	0.9991	0.9991
	χ^2^	0.007	0.02	0.03	0.07
	SSE	0.07	0.42	1.48	5.96
	ARE (%)	0.89	10.5	0.90	0.90
Elovich	a (g mg^−1^)	1.21 ± 0.08	0.61 ± 0.07	0.34 ± 0.08	0.14 ± 0.03
	b (mg g^−1^ min^−1^)	1483 ± 214	7926 ± 347	14,523 ± 1347	25,523 ± 2436
	R^2^	0.9653	0.9657	0.9684	0.9653
	χ^2^	1.11	2.20	7.87	9.63
	SSE	3.35	12.99	38.56	248.2
	ARE (%)	15.77	15.66	21.32	15.75
Avrami	k_AV_ (min^−1^)	1.63 ± 0.11	1.63 ± 0.09	1.62 ± 0.08	1.63 ± 0.07
	q_AV_ (mg g^−1^)	10.33 ± 0.73	20.45 ± 0.87	44.53 ± 1.73	89.04 ± 3.56
	n_AV_	1.12	1.12	1.11	1.12
	R^2^	0.9916	0.9912	0.9914	0.9915
	χ^2^	0.07	0.17	0.34	0.68
	SSE	0.78	3.24	14.88	58.93
	ARE (%)	13.38	14.58	13.41	13.39
General-order	k_n_ (min^−1^ (g mg^−1^) n^−1^)	0.0005 ± 0.0001	0.0005 ± 0.0001	0.0006 ± 0.0001	0.0004 ± 0.0001
	q_n_ (mg g^−1^)	10.70 ± 0.81	21.11 ± 0.78	44.21 ± 1.64	91.65 ± 4.15
	n	4.42	3.85	3.51	3.17
	R^2^	0.9998	0.9996	0.9996	0.9997
	χ^2^	0.001	0.006	0.008	0.02
	SSE	0.01	0.13	0.15	2.06
	ARE (%)	0.30	0.42	0.63	0.43

**Table 5:** The thermodynamic parameters for the dye adsorption on the adsorbent obtained from hart’s-tongue fern (*Asplenium scolopendrium*) leaves.

ΔG^0^ (kJ mol^−1^)			ΔH^0^ (kJ mol^−1^)	ΔS^0^ (J mol^−1^ K^−1^)
285 K	295 K	319 K		
−21.75	−22.65	−24.78	0.44	10.74

**Table 6:** Controllable factors and their levels used to realize the L27 orthogonal array experimental design.

Factor	Level 1	Level 2	Level 3
pH	2	6	12
Time (min)	1	20	60
Adsorbent dose (mg L^−1^)	1	3	6
Initial dye concentration (mg L^−1^)	25	100	200
Temperature (K)	285	295	319
Ionic strength (mol L^−1^)	0	0.10	0.25

**Table 7:** The L27 orthogonal array experimental design, the experimental value obtained for dye removal efficiency, and corresponding S/N ratios after each run.

pH	Time	Adsorbent Dose	Initial Dye Concentration	Temperature	Ionic Strength	Removal Efficiency	S/N Ratio
2	1	1	25	285	0.00	47.63	33.55
2	1	1	25	295	0.10	47.74	33.57
2	1	1	25	319	0.25	48.12	33.64
2	20	3	100	285	0.00	63.48	36.05
2	20	3	100	295	0.10	63.62	36.07
2	20	3	100	319	0.25	64.12	36.13
2	60	6	200	285	0.00	65.71	36.35
2	60	6	200	295	0.10	65.85	36.37
2	60	6	200	319	0.25	66.38	36.44
6	1	3	200	285	0.10	74.12	37.39
6	1	3	200	295	0.25	74.06	37.39
6	1	3	200	319	0.00	77.31	37.76
6	20	6	25	285	0.10	83.22	38.40
6	20	6	25	295	0.25	83.15	38.39
6	20	6	25	319	0.00	86.80	38.77
6	60	1	100	285	0.10	83.68	38.45
6	60	1	100	295	0.25	83.61	38.44
6	60	1	100	319	0.00	87.28	38.81
12	1	6	100	285	0.25	77.74	37.81
12	1	6	100	295	0.00	80.44	38.10
12	1	6	100	319	0.10	81.33	38.20
12	20	1	200	285	0.25	88.45	38.93
12	20	1	200	295	0.00	91.53	39.23
12	20	1	200	319	0.10	92.54	39.32
12	60	3	25	285	0.25	87.47	38.83
12	60	3	25	295	0.00	90.51	39.13
12	60	3	25	319	0.10	91.51	39.22

**Table 8:** Response table for signal-to-noise S/N ratios (larger is better) and the ANOVA results.

Level	pH	Time	Adsorbent Dose	Initial Dye Concentration	Temperature	Ionic Strength
1	35.36	36.38	37.11	37.06	37.31	37.53
2	38.20	37.93	37.56	37.57	37.41	37.45
3	38.76	38.01	37.65	37.69	37.59	37.34
Delta	3.40	1.62	0.54	0.63	0.28	0.19
**Rank**	**1**	**2**	**4**	**3**	**5**	**6**
**Contribution (%)**	75.84	19.04	1.91	2.53	0.46	0.22

## Data Availability

All the experimental data obtained are presented in the form of tables and/or figures, in the article, and in the Supplementary Materials.

## Supplementary Materials

The following supporting information can be downloaded at: https://www.mdpi.com/article/10.3390/polym15193923/s1, Table S1: The non-linear equations of the adsorption isotherms and kinetic models used to assess the adsorption process; Table S2: The calculation equations for error parameters R^2^, χ^2^, SSE, and ARE; Table S3: The equations of specific thermodynamic parameters; Table S4: The equation used to calculate the desorption efficiency; Figure S1: The particle size distribution of hart’s-tongue fern (*Asplenium scolopendrium*) leaves powder; Figure S2: Plot of ln K_L_ vs. 1/T for the dye adsorption onto hart’s-tongue fern (*Asplenium scolopendrium*) leaves powder.
