# Supplementary material for: Utilizing Novel Lignocellulosic Material from Hart’s-Tongue Fern (Asplenium scolopendrium) Leaves for Crystal Violet Adsorption: Characterization, Application, and Optimization
Source: Polymers (Basel). 2023 Sep 28;15(19):3923. doi: 10.3390/polym15193923 (PMC10575424; doi:10.3390/polym15193923)
Supplement: Supplementary file 1 [file polymers-15-03923-s001.zip › polymers-2614632-supplementary.pdf]

polymers

Supplementary material

Utilizing Novel Lignocellulosic Material from Hart's-Tongue Fern (*Asplenium scolopendrium*) Leaves for Crystal Violet Adsorption: Characterization, Application, and Optimization

Giannin Mosoarca, Cosmin Vancea, Simona Popa, Mircea Dan * and Sorina Boran *

Faculty of Industrial Chemistry and Environmental Engineering, Politehnica University Timisoara, Bd. V. Parvan, No. 6, 300223 Timisoara, Romania; giannin.mosoarca@upt.ro (G.M.); cosmin.vancea@upt.ro (C.V.); simona.popa@upt.ro (S.P.)
* Correspondence: mircea.dan@upt.ro (M.D.); sorina.boran@upt.ro (S.B.); Tel.: +40-256404176 (M.D.); +40-256-404-228 (S.B.)

Table S1. The non-linear equations of the adsorption isotherms and kinetic models used to assess the adsorption process.

Adsorption isotherm	Equation
Langmuir isotherm	$q_e = \frac{q_m \cdot K_L \cdot C_e}{1 + K_L \cdot C_e}$
Freundlich isotherm	$q_e = K_F \cdot C_e^{1/n_F}$
Temkin isotherm	$q_e = \frac{R \cdot T}{b} \cdot ln(K_T \cdot C_e)$
Sips isotherm	$q_e = \frac{Q_{sat} \cdot K_S \cdot C_e^n}{1 + K_S \cdot C_e^n}$
Redlich-Peterson isotherm	$q_e = \frac{K_{RP} \cdot C_e}{1 + a_{RP} \cdot C_e^{\beta_{RP}}}$
**Kinetic model**	**Equation**
Pseudo-first-order kinetic model	$q_t = q_e(1 - exp^{-k_1 \cdot t})$
Pseudo-second-order kinetic model	$q_t = \frac{k_2 \cdot t \cdot q_e^2}{1 + k_2 \cdot t \cdot q_e}$
Elovich kinetic model	$q_t = \frac{1}{a} ln(1 + a \cdot b \cdot t)$
Avrami kinetic model	$q_t = q_{AV}[1 - exp(-k_{AV} \cdot t)^{n_{AV}}]$
General order kinetic model	$q_t = q_n - \frac{q_n}{[k_n \cdot (q_n)^{n-1} \cdot t \cdot (n-1) + 1]^{1/1-n}}$

where:

- q_m and Q_{sat} are the maximum absorption capacities; K_L, K_F, K_T, K_S and K_{RP} are the Langmuir, Freundlich, Temkin, Sips and Redlich-Peterson isotherms constants; $1/n_F$ is an empirical constant indicating the intensity of adsorption; R is the universal gas constant; T is the absolute temperature; b is Temkin constant which related to the adsorption heat; n is Sips isotherm exponent; a_{RP} is Redlich-Peterson isotherm constant and β_{RP} is Redlich-Peterson exponent which can vary between 0 and 1.

- q_t is the dye amount adsorbed at time t; k_1, k_2, k_n and k_{AV} are the rate constants of pseudo-first-order, pseudo-second-order, general order and Avrami kinetic models; q_e, q_n and q_{AV} are the theoretical values for the adsorption capacity; a is the desorption constant of

Elovich model; b is the initial velocity; n is the general order exponent and n_{AV} is a fractional exponent.

Table S2. The calculation equations for error parameters R^2, χ^2, SSE and ARE.

Function name	Equation
Determination coefficient (R^2)	$R^2 = 1 - \frac{\sum_{i=1}^{n} \left(y_{i,exp} - y_{i,mod}\right)^2}{\sum_{i=1}^{n} \left(y_{i,exp} - \overline{y_{i,exp}}\right)^2}$
Sum of square error (SSE)	$SSE = \sum_{i=1}^{n} \left(y_{i,exp} - y_{i,mod}\right)^2$
Chi-square (χ^2)	$\chi^2 = \sum_{i=1}^{n} \frac{\left(y_{i,exp} - y_{i,mod}\right)^2}{y_{i,mod}}$
Average relative error (ARE)	$ARE = \frac{100}{n} \sum_{i=1}^{n} \left\lvert \frac{y_{i,exp} - y_{i,mod}}{y_{i,mod}} \right\rvert$

where: $y_{i,exp}$ is the experimental value; $y_{i,mod}$ is the modeled value; $\overline{y_{i,exp}}$ is the mean values and n is the total amount of information.

Table S3. The equations of specific thermodynamic parameters.

Thermodynamic parameters	Equation
Standard Gibbs free energy change	$\Delta G^0 = -RT \ln K_L$
Standard enthalpy change<br>Standard entropy change	$\ln K_L = \frac{\Delta S^0}{R} - \frac{\Delta H^0}{RT}$

where: R represents the universal gas constant; K_L represents the Langmuir constant, T represents the absolute temperature.

Table S4. The equation used to calculate the desorption efficiency

Desorption efficiency equation
$D(\%) = \frac{m_d}{m_a} \cdot 100$

where: m_d is the crystal violet amount liberated by the desorbing agent, m_a is the crystal violet amount adsorbed on the adsorbent material.

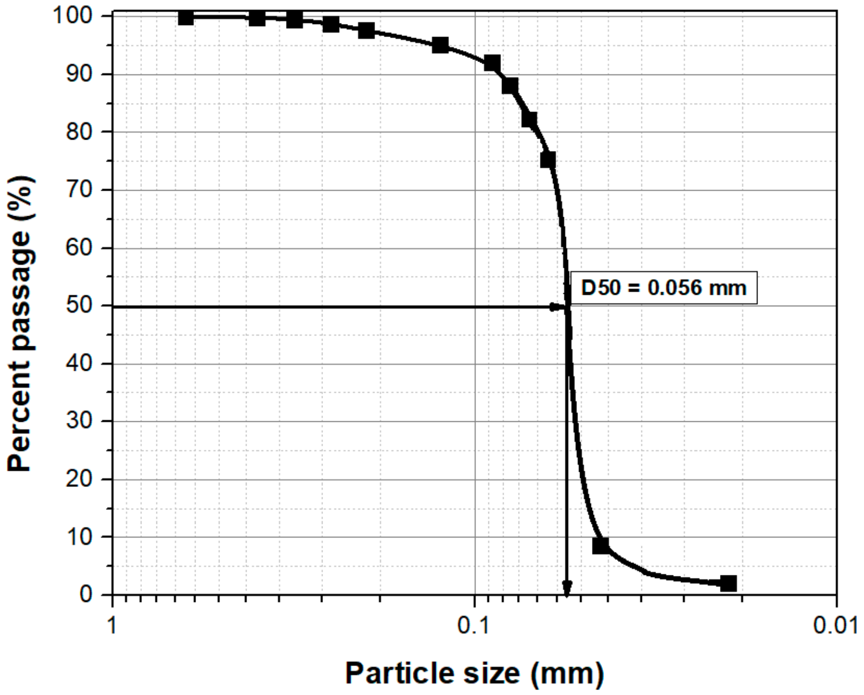

Figure S1. The particle size distribution of hart's-tongue fern (*Asplenium scolopendrium*) leaves powder

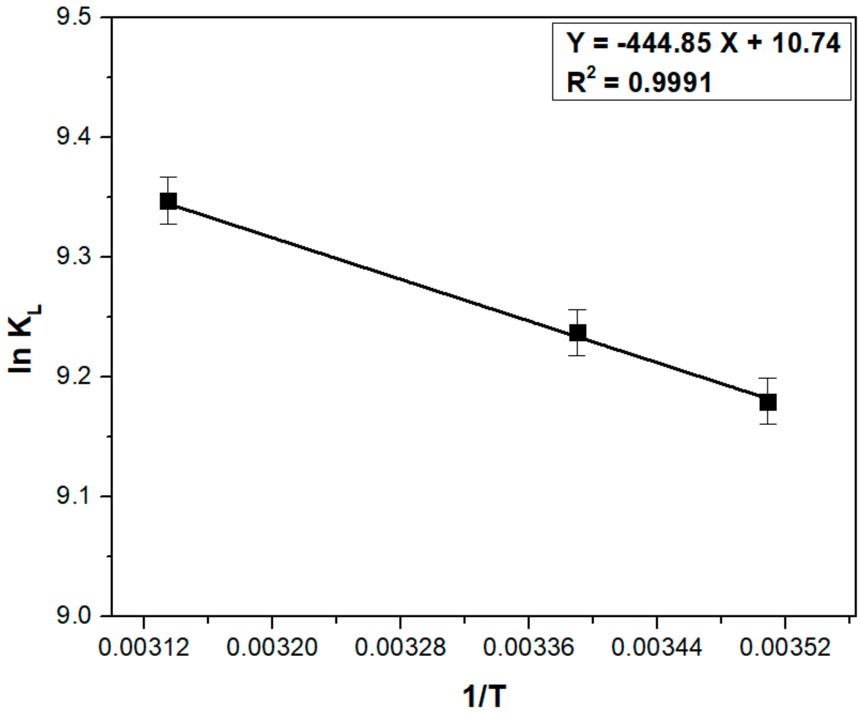

Figure S2. Plot of ln K_L vs. 1/T for the dye adsorption onto hart's-tongue fern (*Asplenium scolopendrium*) leaves powder
